# Magnetic resonance of the NiFe_2_O_4_ nanoparticles in the gigahertz range

**DOI:** 10.1186/1556-276X-8-404

**Published:** 2013-10-01

**Authors:** Zhenhua Shi, Jing Zhang, Daqiang Gao, Zhonghua Zhu, Zhaolong Yang, Zhipeng Zhang, Desheng Xue

**Affiliations:** 1 Key Laboratory for Magnetism and Magnetic Materials of MOE, Lanzhou University, Lanzhou 730000, People’s Republic of China

**Keywords:** NiFe_2_O_4_ nanoparticles, Sol–gel method, High-frequency magnetic resonance

## Abstract

We report an adjustable magnetic resonance frequency from 1.45 to 2.54 GHz for NiFe_2_O_4_ nanoparticles which were prepared by a sol–gel process. X-ray diffraction and scanning electron microscopy results indicate that the samples are polycrystalline nanoparticles, and the size of the particles increases obviously with the thermal treatment temperature. The consequence of the surface composition suggests that the oxygen defects are present in the nanoparticle surface, and this surface magnetic state can show a strong surface anisotropy. With decreasing size of the particle, the surface magnetic effect is predominant, resulting in an increase of resonance frequency for NiFe_2_O_4_ nanoparticles. This finding provides a new route for NiFe_2_O_4_ materials that can be used in the gigahertz range.

## Background

Soft magnetic ferrites have attracted much attention in recent years because they have large saturation magnetization (*M*_s_), low electrical conductivity, and excellent chemical stabilities [1,2] and can be used as ferrofluids [3], in magnetic resonance imaging [4], and in microwave devices [5,6]. Furthermore, nanoscale soft magnetic ferrites exhibit special magnetic-like, magneto-resistive, and magneto-optical properties compared with bulk magnetic materials [7]. Because the surface-to-volume ratio becomes very large with the reduction of the particle size at nanoscale, they are potentially useful for a broad range of applications. Soft magnetic ferrites have a potential application in electronic devices when used in the gigahertz (GHz) range. This is because in this frequency region, magnetic metals exhibit strong eddy current loss [8] compared to soft magnetic ferrites [9,10]. For soft magnetic ferrites, there is magnetic resonance, resulting in magnetic losses. This provides some limitations (like threshold frequency) of the application. Nakamura [11] and Tsutaoka et al. [12] reported that the resonance frequency of bulk soft magnetic ferrites is much lower than 1 GHz. It seems to be an urgent issue to improve the resonance frequency of soft magnetic ferrites. Guo et al. reported that Ni-Zn ferrite thin films exhibit much higher natural resonance frequency, thanks to bianisotropy [13]. There is strong surface anisotropy in ferrite nanoparticles (NPs), which has been reported before [14-16]. Owing to this surface anisotropy, ferrite NPs will likely show high resonance frequency. NiFe_2_O_4_ is a typical soft magnetic ferrite with high electrical resistivity [17], and it is an inverse spinel with metal ions occupying the octahedral and tetrahedral sites. The magnetic moments placed in the tetrahedral site and octahedral site couple in an antiparallel manner by a superexchange interaction which is mediated through adjacent oxygen atoms and forms a collinear ferrimagnetic ordering. Additionally, the magnetic behaviors of nanoscale NiFe_2_O_4_ are extremely sensitive to their size [18]. There is already a significant interest in synthesizing NiFe_2_O_4_ NPs for achieving optimal magnetic properties [19-21]. In this work, NiFe_2_O_4_ NPs were prepared using the sol–gel method. The morphology, structure, and magnetic characterization of the NiFe_2_O_4_ NPs have been systemically investigated. Importantly, an adjustable magnetic resonance has been observed in the GHz range, implying that NiFe_2_O_4_ is a candidate for microwave devices in the GHz range.

## Methods

NiFe_2_O_4_ NPs were synthesized by the sol–gel method with a postannealing process [22]. All chemical reagents used as starting materials are of analytical grade and purchased without any further treatment. In a typical synthesis process, 0.01 M Ni(NO_3_)_4_·5H_2_O, 0.02 M Fe(NO_3_)_3_·9H_2_O, and 0.03 M citric acid were firstly dissolved in 100 ml of deionized water. The molar ratio of metal ions to citric acid was 1. A small amount of ammonia was added to the solution to adjust the pH value at about 7 with continuous stirring. Then, the dissolved solution was stirred for 5 h at 80°C and dried in the oven to form the precursor at 140°C. The precursor was preannealed at 400°C for 2 h and then calcined at different temperatures (700°C, 800°C, 900°C, and 1,000°C) for 2 h in the air, which were denoted as S700, S800, S900, and S1000, respectively.

X-ray diffraction (XRD; X'Pert PRO PHILIPS with Cu Kα radiation, Amsterdam, The Netherlands) was employed to study the structure of the samples. The morphologies of the samples were characterized using a scanning electron microscope (SEM; Hitachi S-4800, Tokyo, Japan). The measurements of magnetic properties were made using a vibrating sample magnetometer (VSM; LakeShore 7304, Columbus, OH, USA). The chemical bonding state and the compositions of the samples were determined by X-ray photoelectron spectroscopy (XPS; VG Scientific ESCALAB-210 spectrometer, East Grinstead, UK) with monochromatic Mg Kα X-rays (1,253.6 eV). The complex permeability *μ* of the particles/wax composites were measured on a vector network analyzer (PNA, E8363B, Agilent Technologies, Inc., Santa Clara, CA, USA). Then, 63 vol.% of particles and 37 vol.% of wax were mixed together and pressed into a coaxial cylindrical specimen, in which the magnetic particles were randomly dispersed. Electron spin resonance (ESR) measurements were performed with a Bruker ER200D spectrometer (JEOL, Tokyo, Japan).

## Results and discussion

**Figure 1:**
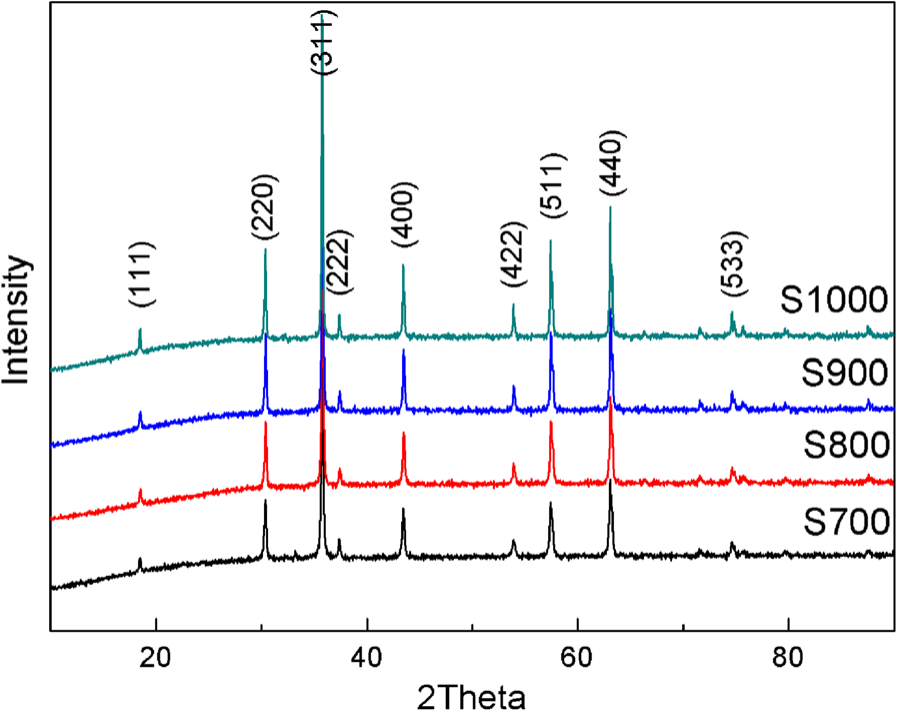
X-ray diffraction patterns for samples S700, S800, S900, and S1000.

**Figure 2:**
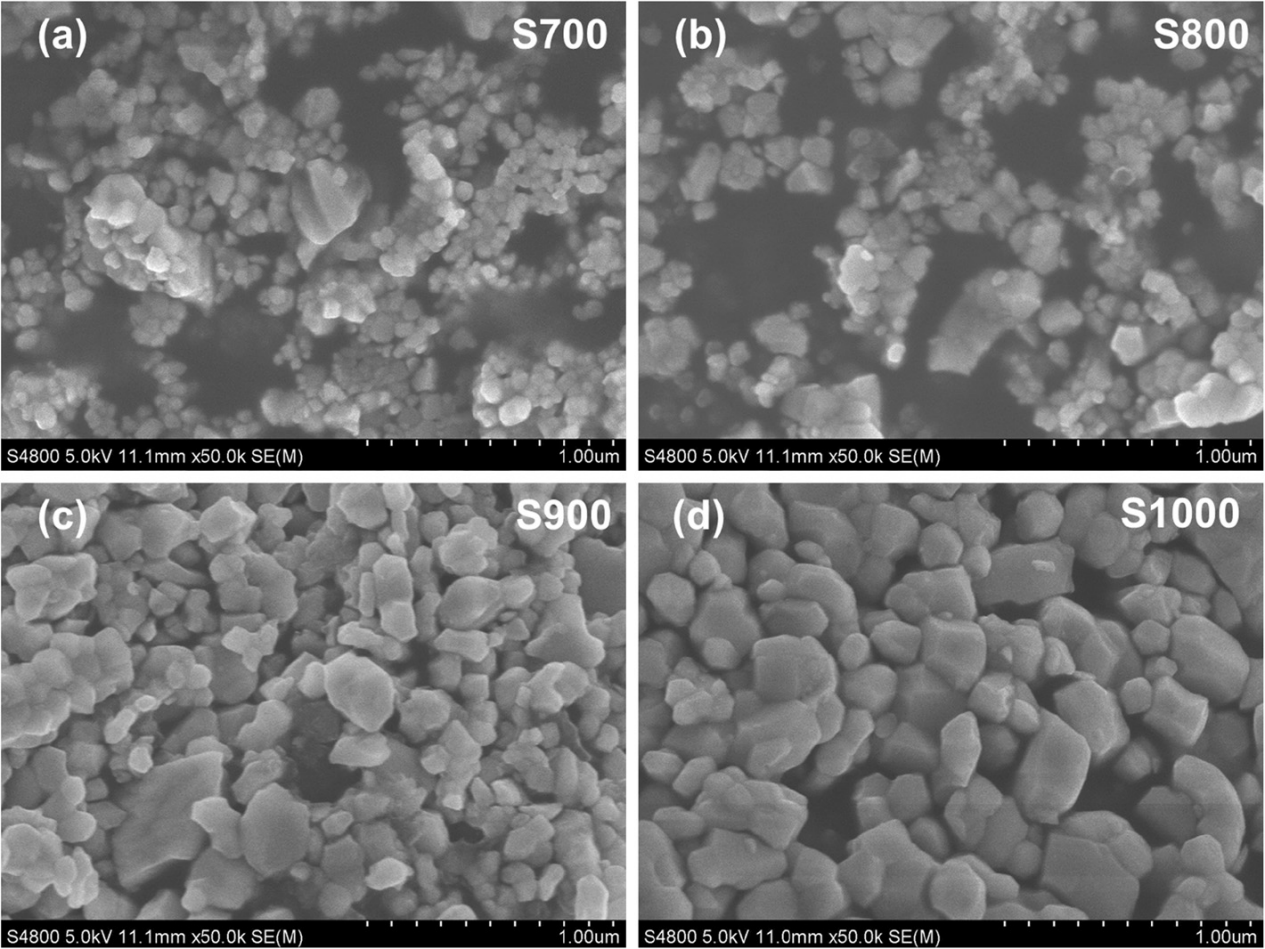
SEM images of samples S700 (a), S800 (b), S900 (c), and S1000 (d).

The XRD patterns of NiFe_2_O_4_ NPs annealed at 700°C to 1,000°C for 2 h are depicted in Figure 1. All diffraction peaks of the samples can be well indexed to the standard spinel phase without any additional peak. The average crystallite size of the synthesized powders is estimated by the X-ray peak broadening of the (400) diffraction peak, via the Scherrer equation [23]. The results indicate that the powders are nanocrystalline with an average crystallite size of 31 to 46 nm for S700 to S1000. Figure 2a,b,c,d shows the SEM images of NiFe_2_O_4_ NPs. It is clearly seen that all the NiFe_2_O_4_ NPs are partly accumulated together with different sizes, and the size of the sample particles increases obviously with the thermal treatment temperature. The average particle size is about 60 nm for S700 (200 nm for S1000), which is much larger than the crystallite size estimated by XRD. These results indicate that the obtained sample particles are polycrystalline.

**Figure 3:**
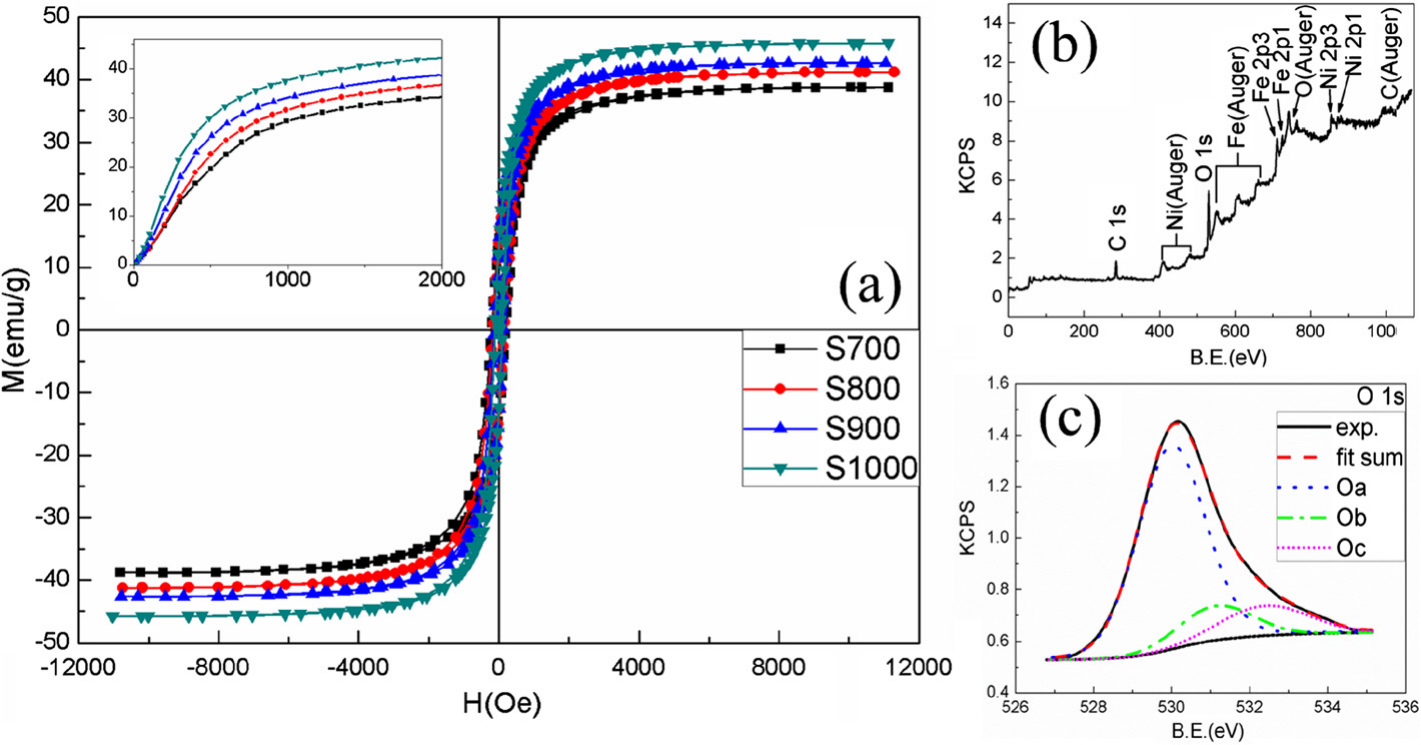
***M*-*H *curves of the samples and XPS spectra of S700. (a)** Magnetic hysteresis loops of the samples (inset: the initial magnetization curves), **(b)** XPS survey spectrum of sample S700, and **(c)** fitted XPS spectra of O 1*s* of sample S700. The vertical axis represents the signal intensity. KCPS, kilo counts per second; B.E., binding energy.

The room temperature magnetic properties of NiFe_2_O_4_ NPs were studied using VSM. Figure 3a shows the hysteresis loops of the samples, and the inset of Figure 3a shows the initial magnetization curves. It is found that *M*_s_ is a monotonic function of the annealing temperature, and the value of *M*_s_ is 38.7, 41.1, 42.6, and 45.8 emu/g for S700 to S1000, respectively. Generally, the *M*_s_ of NiFe_2_O_4_ NPs is lower than that of the bulk form (56 emu/g) [24,25], which can be attributed to the greater fraction of surface spins in NPs that tend to be canted or the spin disorder with a smaller net moment [26]. The spin disorder is due to the presence of considerable defects which can destroy the superexchange interaction. *M*_s_ increases as the sintering temperature increases, which is due to the reduction of the specific surface area. The initial magnetization curves suggest that the initial magnetic permeability increases with increasing annealing temperature.

The evidence for the composition of products in the surface was obtained by XPS. Figure 3b shows the XPS survey scan spectrum of a representative sample, S700, indicating that no impurities were detected in the sample within the detection limit. An asymmetric high-resolution XPS spectrum for the O 1*s* peak is observed (shown in Figure 3c), which has a shoulder at the higher binding energy side. By fitting, we obtained three peaks at 529.8, 531.2, and 532.4 eV. The dominant peak located at 529.8 ± 0.2 eV (Oa), which corresponds to O^2−^ ions of the pure composites [27,28], and the highest binding energy peak at 532.4 ± 0.2 eV (Oc) can be attributed to the chemisorbed oxygen of surface hydroxylation, oxygen atoms in carbonate ions, and adsorbed H_2_O or O_2_[29]. Furthermore, the medium binding energy component (Ob) located at 531.2 ± 0.2 eV (Oc) is associated with the O^2−^ ions in the oxygen-deficient regions (O vacancies) [30]. The result obviously demonstrates the presence of oxygen defects in the surface, and the oxygen defects can destroy the superexchange interaction. This indicates that surface and internal magnetic states are different, and the surface magnetic state can show a strong surface anisotropy [14].

**Figure 4:**
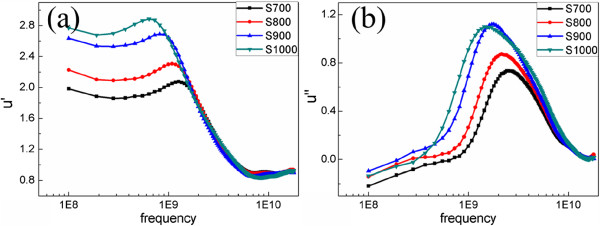
**Effective complex permeability *μ *of the samples. (a)** Spectra of the real part (*μ'*_eff_). **(b)** Spectra of the imaginary part (*μ''*_eff_).

Figure 4 shows the complex permeability *μ* of the NiFe_2_O_4_/wax with 63 vol.%. At a frequency of 0.1 GHz, the real part of the complex permeability (*μ'*; Figure 4a) increases from 2.0 to 2.8 with the increase of sintering temperature. The spectra of the imaginary part (*μ''*) are shown in Figure 4b; it is worth noting that a resonance phenomenon in the effective permeability is observed at around 1 ~ 3 GHz for NiFe_2_O_4_ NPs. Meanwhile, with the increase of sintering temperature, continuous modification in the resonance frequency of the samples in the range of 1.45 to 2.54 GHz has been achieved, which is much higher than previously reported [31]. Pascard and Globus reported that the magnetic resonance frequency is approximately 10^2^ MHz for NiFe_2_O_4_ microparticles [32]. Based on the Landau-Lifshitz-Gilbert equation, the resonance frequency is *f*_r_ = (1 + *α*^2^) × *γ* × *H*_*a*_*/*2*π* (*α* is the magnetic damping parameter, *γ* is the gyromagnetic ratio, *H*_*a*_ is the magnetic effective anisotropy field), and Vittoria et al. reported that *α* is less than 0.01 [33]. As a result, an approximately effective anisotropy field is 900, 760, 610, and 510 Oe for S700, S800, S900, and S1000, respectively. The data unambiguously show that the magnitude of the effective anisotropy field is on the decline with the increase of sintering temperature. For NiFe_2_O_4_ NPs, a strong effective anisotropy has been obtained, which is consistent with previous theoretical results [14-16]. This effective anisotropy field is much bigger than the magnetocrystalline anisotropy field for NiFe_2_O_4_; therefore, it is related to the strong surface anisotropy for NPs. The magnitude of this surface anisotropy is related to the concentration of the defects in the surface and the fraction of broken exchange bonds relative to the total number of neighboring pairs of surface cations [14], for an individual particle. When the NiFe_2_O_4_ particles gradually grow, as the sintering temperature increases, the specific surface area decreases while the defects in the surface are also reduced. This weakens the surface anisotropy and then reduces the resonance frequency.

**Figure 5:**
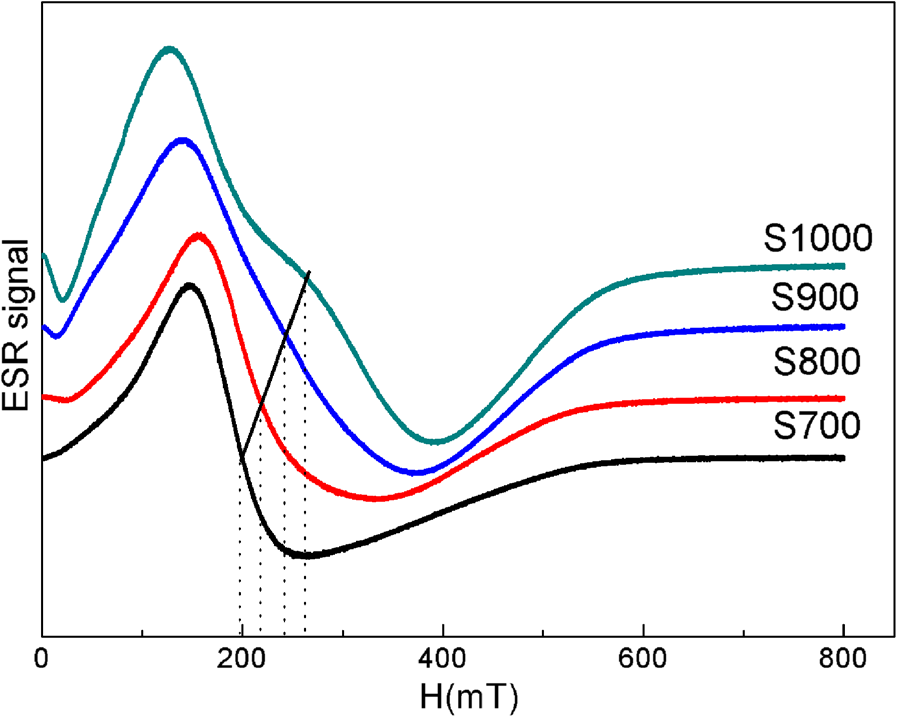
ESR spectra of samples.

In order to further identify this magnetic resonance, ESR measurement was performed. The results for the samples are displayed in Figure 5. It can be seen that all the samples show an obvious ferromagnetic resonance, and the resonance field is proportional to the sintering temperature. The particle diameter is directly proportional to the sintering temperature as can be seen from Figure 2. This behavior can be explained by the core-shell morphology of the NPs consisting of ferrimagnetically aligned core spins and the surface in which part of the superexchange interaction is destroyed. The magnetic behavior of the NPs has a marked dependence on the particle size, and the surface effects start to dominate as the particle size decreases. *g*_eff_ is the effective *g*-factor introduced by analogy with the Lande *g*-factor and calculated via *g*_eff_ = *hν* / *μ*_B_*H*_*r*_[34], where *h* is the Planck constant, *ν* is the microwave frequency, *μ*_B_ is the Bohr magneton, and *H*_*r*_ is the resonance field. Fe^3+^ ions usually exhibit two well-defined signals of *g*_eff_ = 2.0 and 4.3; the signal of *g*_eff_ = 4.3 has been ascribed to the isolated Fe^3+^ ions, while the signal of *g*_eff_ = 2.0 has been assigned to the Fe^3+^-coupled pair (Fe^3+^-O-Fe^3+^) [35]; Ni^2+^ ions normally show *g*_eff_ values of 2.2 and 2.0, corresponding to the Ni^2+^-coupled pair (Ni^2+^-O-Ni^2+^) and the isolated Ni^2+^ ions, respectively [36,37]. The value of *g*_eff_ characterizing polycrystalline NiFe_2_O_4_ is 2.4 as reported before [35]. As can be seen from Figure 5, *g*_eff_ is gradually decreasing as the sintering temperature increases. For S700, the ESR spectrum exhibits a large *g*_eff_ of 3.19 corresponding to the low *H*_*r*_. This is because, first, there is a dipole interaction between the magnetic moments of the neighboring metal ions which destroys the superexchange interaction between them and leads to the strong surface anisotropy [14]. Second, the internal magnetic moment is coupled to the magnetic moment in the surface, and the sample shows a low *H*_*r*_, when the size of particles is small enough. In contrast, when the size of particles increases, the internal magnetic state becomes independent of the surface, owing to a finite exchange interaction length. Therefore, sample S1000 exhibits two resonance peaks. This is the further evidence of our previous inference.

## Conclusions

In summary, NiFe_2_O_4_ NPs were obtained using the sol–gel method, and the magnetic properties of NiFe_2_O_4_ NPs regularly change with the sintering temperature. Notably, NiFe_2_O_4_ NPs exhibit magnetic resonance in the GHz range. Through the study of the surface composition, the presence of oxygen defects, which can destroy the superexchange interaction, in the surface can be deduced. The strong surface anisotropy field for NiFe_2_O_4_ NPs has an effect on the core of particles, and this leads to a strong effective anisotropy field, thereby generating high-frequency magnetic resonance for NiFe_2_O_4_. ESR spectra measured at room temperature further confirm that surface magnetism plays a great role.

## Competing interests

The authors declare that they have no competing interests.

## Authors’ contributions

ZS prepared all the samples, participated in all the measurements and data analysis, and drafted the manuscript. DX and DG conceived and designed the manuscript. JZ carried out the XPS measurements and data analysis. ZZ1 carried out the XRD measurements and data analysis. ZZ2 participated in the VSM measurements. ZY participated in the data analysis and interpretation of the results. All authors have been involved in revising the manuscript and read and approved the final manuscript.
